# Knowledge Level of the Primary Healthcare Providers on Chronic Obstructive Pulmonary Disease and Pulmonary Rehabilitation

**DOI:** 10.1155/2015/538246

**Published:** 2015-10-26

**Authors:** Tuğba Göktalay, Ayşe Nur Tuncal, Seçil Sarı, Galip Köroğlu, Yavuz Havlucu, Arzu Yorgancıoğlu

**Affiliations:** ^1^Department of Pulmonology, Celal Bayar University, Manisa, Turkey; ^2^Public Health Directorate, Manisa, Turkey

## Abstract

*Introduction*. Awareness of the healthcare providers on chronic obstructive pulmonary disease (COPD), which is an important cause of mortality and morbidity in our country and all over the world, and on pulmonary rehabilitation (PR) which plays an important role in its nonpharmacological treatment will provide effectiveness in diagnosis and treatment of COPD. The present study aimed at determining knowledge level of the healthcare providers about COPD and PR. *Materials and Methods*. In this cross-sectional study, family practitioners and staff of home-care in central county of Manisa City were applied a questionnaire in order to determine their knowledge level on COPD and pulmonary rehabilitation during the in-service training on “pulmonary rehabilitation, home-care services for the pulmonary diseases, and respiratory exercises.” *Results*. 65.5% of the healthcare providers responded to the survey. Rate of those correctly knowing at least one of four items was 97.2%. No responder knew all items correctly. Average value for correct answers was 5.30 ± 2.1 (range: 1–10). The physicians, men, and those working in family health centers had higher level of knowledge on COPD compared to nonphysician healthcare providers (*p* = 0.006), women (*p* = 0.002), and those working in other practices (*p* = 0.019), respectively. *Conclusion*. Knowledge level of the primary healthcare providers on COPD and PR remains inadequate. Dynamic postgraduate training on this topic will be useful in referring the patients to centers giving service for this condition.

## 1. Introduction

Chronic obstructive pulmonary disease (COPD) is an important cause of mortality and morbidity in our country and all over the world. Prevalence of COPD is being anticipated to increase due to continuing risk factors and aging of the society [1]. Being a preventable and manageable condition, CODP ranks fourth in our country and third on the world in the list of causes of death [1, 2]. Main reasons for low rate of making diagnosis are that the individuals realize the symptoms late or the symptoms appear late in the course of the disease as well as low utilization of respiratory function test and uncertainty of the diagnostic criteria.

Pulmonary rehabilitation (PR) is a multidisciplinary, evidence-based therapeutic approach to the patient used in management of the symptoms that is specific to the patient. Its main objectives are to decrease the symptoms, to increase quality of life and contribution to the daily life by optimizing the emotional status, and to reduce health-related expenses by reversing or stabilizing the systemic effects of the disease [1]. It has been used in treatment and management of the patients with COPD since the 1990s [3]. PR improves sense of dyspnea, quality of life, exercise capacity, hospital admission rate, anxiety, and depression in the patients with COPD at the level A. It has also positive effects on mortality [4, 5].

Although COPD is a disease involving the respiratory medicine experts, its symptoms and diagnostic criteria should also be known by the primary healthcare providers considering its economical burden. Early diagnosis of the disease and having the patients quit smoking, the most significant etiological factor, are important in taking measures prior to proceeding to advanced stages of the disease requiring symptomatic and expensive approaches. COPD, however, is not known adequately and underdiagnosed and undertreated [1, 2], being compatible with data from the studies from our country and world [6–8]. In the Turkish Pilot Study BOLD on COPD conducted in Adana, prevalence of COPD in the individuals older than 40 years was 19.1% while rate of the patients with COPD diagnosed by a physician was found to be 5.6% [9].

In Manisa City, in-service training was made titled “pulmonary rehabilitation, home-care services in the respiratory diseases, and respiratory exercises” on March 13, 2014, in corporation with Directorate of Public Health and Department of Chest Diseases of Medical School of Celal Bayar University. The training was performed for the family physicians and home-care service providers practicing in Manisa City. Prior to this training program, pretest was applied in order to determine knowledge level of the participants on COPD and PR. Research hypothesis was to be low knowledge level of the family physicians and home-care service (HCS) providers on COPD and PR. Aim of the present cross-sectional study was to determine knowledge level of the family physicians and home-care service (HCS) providers on COPD and PR.

## 2. Materials and Methods

In Manisa City, in-service training was made titled “pulmonary rehabilitation, home-care services in the respiratory diseases, and respiratory exercises” on March 13, 2014. This training activity was conducted in corporation with Directorate of Public Health and Department of Chest Diseases of Medical School of Celal Bayar University.

Prior to the training program given in two separate seasons, a survey was applied to the participants consisting of 5 items on sociodemographical characteristics and 14 items on knowledge level on the COPD and PR (Appendices A and B). The survey was used which was developed by Study Group 2 developing and administering the strategic plan in the context of the target “advocating and making the public adopt the program and the diseases” of the Action Plan and Control Program for Preventing Chronic Airway Diseases [6].

Knowledge level of the participants was evaluated based on age, sex, occupation, working years, and practice settings.

The data were analyzed by frequency and chi-square tests using SPSS software v.15.0. Chi-square test was used to compare the answers to the items based on sex, age, working years, occupational group, and practice settings. *p* values less than 0.05 were considered as statistically significant.

## 3. Results

The sample of the study included 130 individuals with 110 being invited to participate in the study. 65.5% of the participants in the training program responded to this cross-sectional study.

Of the participants to the training 72.7% were practitioners, 9.7% were specialist physicians, and 18.1% were other types of healthcare providers (nurses, dentists, and health officers). Of the participants, 35.9% were female and 64.1% were male; mean age was 45.6 ± 7.1 years (range: 25–61 years); mean number of working years was 21.4 ± 6.6 (range: 1–32 years). Of the participants, 80.0% were practicing in family health center (FHC), 12.9% in home-care services (HCS), and 7.1% in the center of oral and dental health (CODH) (Table 1).

Rate of correctly knowing at least one of four items to measure knowledge level of the responders was 97.2%. No responder knew all items correctly. Average value for correct answers was 5.30 ± 2.1 (range: 1–10). The physicians, men, and those working in family health centers had higher level of knowledge on COPD compared to nonphysician healthcare providers (*p* = 0.006), women (*p* = 0.002), and those working in other practices (*p* = 0.019), respectively.

Rate of correctly knowing the items related to knowledge level, consisting of 6 items, is given below along with frequency distribution based on sections and questions. Sociodemographical characteristics were compared to knowledge level and those found to be significant were presented separately (Table 2).

The items in the first section (questions (1) and (2)) were related to definition, risk factors, pathophysiology, and epidemiology of COPD. Rate of correctly knowing 2 items in this section was 33.3% (*n* = 24) with those older than average age (*p* = 0.022) and those with more working years (>21.4 years) (*p* = 0.008) correctly answered this item. 73.9% of the participants correctly answered the item “what are the risk factors for COPD,” which was one of the two items. 51.5% of the participants correctly answered the second item noting that “COPD ranks third in causes of death in Turkey” with men (*p* = 0.009), elders (being more than 45.6 years old) (*p* = 0.019), and those with more working years (>21.4 years) (*p* = 0.022) answering this item correctly.

The items in the second section (questions (3)–(5)) were related to diagnosis and management of COPD. Rate of knowing correctly all three items in this section was 8.3% (*n* = 6). Of the participants, 46.7% correctly knew spirometric description of COPD and 25.4% of them correctly knew that the first method was reversibility test for differential diagnosis for airway obstruction. Of the participants, 49.3% correctly knew differential diagnosis of asthma and COPD with physicians (*p* = 0.045), elder participants (>45.6 years old) (*p* = 0.004), those practicing in FHCs (*p* = 0.043), and those with more working years (>21.4 years) (*p* = 0.001) correctly knew this item.

The items in the third section (question (6)) were related to basic concepts on the PFT for COPD. The only item in this section specified that FEV1 was the concept used with priority in order to assess the response of the patients with COPD to the treatment; 14.1% of the participants (*n* = 9) correctly knew this item. No statistically significant difference was found when the items in this section were evaluated.

The items in the fourth section (questions (7)–(9)) were related to patient education and pharmacological treatment in COPD. Rate of correctly knowing three items in this section was 5.6% (*n* = 4). Of the participants, 85.7% correctly knew that the most important intervention was “quitting smoking and avoiding risk factors”; 26.9% correctly knew that “administration of pneumococcal vaccine” was not necessary to inquire; 25.0% correctly knew that, in managing COPD treatment, pulmonary rehabilitation should be started when expected postbronchodilator FEV1 was <80%. No statistically significant difference was found when the items in this section were evaluated.

The items in the fifth section (questions (10)–(12)) were related to nonpharmacological treatment of COPD. Rate of correctly knowing all three items in this section was 9.7% (*n* = 7). Of the participants, 29.0% correctly knew aims of the pulmonary rehabilitation in COPD; 31.7% correctly knew home-care criteria in COPD; and 49.2% correctly knew headings of evaluating effectivity in the pulmonary rehabilitation. No statistically significant difference was found when the items in this section were evaluated.

The items in the sixth section (questions (13) and (14)) were related to approach to diagnosis and treatment of COPD exacerbation. Rate of correctly knowing all two items in this section was 1.4% (*n* = 1). 34.4% of the participants correctly knew the approach to the treatment for the patient in question. Those with more working years (>21.4 years) correctly answered this item (*p* = 0.024). 27.6% of the participants correctly knew use of antibiotics in exacerbation of COPD.

## 4. Discussion

Being the fourth leading cause of death in our country, COPD is not known adequately and is underdiagnosed. Diagnosis and management of the disease are primarily done by specialists of respiratory diseases. In diagnosis of the disease selection of the patients, quality of staff, evaluation, compliance of the patient, and knowledge of the physician are also important [10]. For fighting the disease, it is also important that primary care physicians and other primary healthcare providers have adequate level of knowledge on risk factors, diagnosis and management, pharmacological and nonpharmacological therapeutic approaches, and approaches to the exacerbation.

In a survey evaluating knowledge level of 139 medical students on COPD, average number of correct answers of the students was found to be 8.35 ± 2.75 and their knowledge level was considered to be intermediate. The researchers noted that education in the medical faculty on COPD should be given meticulously [8]. Walters et al. found that attitudes of the primary care physicians had a role in delay in diagnosing COPD [11]. In a study by Başyiğit et al. evaluating effects of postgraduate training on knowledge level on COPD, the authors found that there was a significant increase in the rate for the physicians practicing in the primary care to correctly answer the questions in the COPD survey and noted that the primary care physicians had lack of information on COPD [12]. In the present study as well, knowledge level of the healthcare providers was low. Mean number of correct answers of the physicians was higher than other healthcare providers although it was still at inadequate level. Increasing level of knowledge by providing in-service training to the physicians and other healthcare providers seems to be important.

Using tobacco and its derivatives is the most important risk factor in development of COPD. Although rate of smoking reduced in our country as a consequence of efforts to fight smoking, it is still at a rate of 27% in the individuals >15 years old [13]. In the study by Yildiz et al., rate of knowing that smoking was the most important risk factor in development of COPD was 51% [6]. In the study by Başyiğit et al., all of 92 physicians practicing in the primary care answered correctly the questions related to the etiological factor [12]. In the present study, rate of accuracy we found about risk factors for COPD among the physicians and other healthcare providers practicing in the primary care was lower. This discordance was mainly attributable to the fact that all participants in the study of Başyiğit et al. were physicians. In the present study, however, accuracy rate was found to be higher in those with more working years.

Unfortunately, rate of using pulmonary function test (PFT) in diagnosing COPD is low [10, 14, 15]. When the second and third sections of our survey related to diagnosis and management of COPD were evaluated, physicians, the elder participants, those practicing in ASMs, and those with more working years were more competent in using PFT although it may be concluded that rate of using and assessing PFT was low.

Being one of the nonpharmacological therapeutic methods for COPD and playing an important role in decreasing COPD symptoms, pulmonary rehabilitation (PR) is not known adequately by the physicians and other healthcare providers in the primary care. Pulmonary rehabilitation programs have been applied by specialists of chest diseases for almost 10 years in our country. In 2014, activities of the week of pulmonary rehabilitation were held for the first time in order to create awareness. Decramer et al. found that the specialist physicians were not attentive in COPD to the pulmonary rehabilitation, using noninvasive mechanical ventilation, and methods to quit smoking [16]. In the present study, in addition to overall lack of adequate information on COPD, rate of correct answers to the questions on nonpharmacological treatment of COPD also remained low. Nonetheless, the questions related to timing and targets of starting pulmonary rehabilitation were correctly answered with rates of 25% and 29%, respectively. Information on methods to evaluate effectiveness of pulmonary rehabilitation such as exercise capacity, quality of life, degree of the symptoms, body composition, and psychosocial status was at higher level. In the present study, however, we found that the variables of age, sex, working years, occupational group, and practice setting had no effect on it. Including nonpharmacological therapeutic approaches in the postgraduate training given to the physicians and other healthcare providers as well as specialist physicians will be useful in creating awareness and referring the patients in need to the centers providing service on this subject.

The fact that rate of responding remained at 65% is one of the limitations of the current study. The main limitation of the present study was that posttraining survey was not applied. Nevertheless, the data we obtained indicate that knowledge level of the physicians and other healthcare providers practicing in primary care on COPD was inadequate. Nonpharmacological treatment of COPD including PR is also not known sufficiently. Our findings support that postgraduate training efforts remain inadequate on COPD. As a consequence of the present study, it was concluded that postgraduate training to the primary care providers should be performed more dynamically on preventing development and progression of the disease and its early detection, effective treatment, and preventing development of complications. It was also concluded that national sources of written and visual media emphasizing importance of physical activity should be used in order to create awareness among the healthcare providers and in the society and that number of the centers giving PR should be increased.

## Figures and Tables

**Table 1:** Sociodemographical characteristics of the responders.

	*n*	%^*∗*^
Sex (*n* = 64)		
Female	23	35,9
Male	41	64,1
Range of age (*n* = 69)		
20–30 years	4	5,8
31–40 years	9	13,0
41–50 years	40	58,0
51–60 years	15	21,7
61+	1	1,5
Average years (*n* = 67)		
Above average years (>45,6)	31	46,3
Below average years (≤45,6)	36	53,7
Working years (*n* = 65)		
1–10 years	5	7,7
11–20 years	21	32,3
21+	39	60,0
Mean working years (*n* = 64)		
Above the average (>21,4)	29	45,3
Below the average (≤21,4)	35	54,7
Occupational group (*n* = 72)		
General practitioner	52	72,2
Specialist physician	7	9,7
Other	13	18,1
Practice setting (*n* = 70)		
FHC	56	80,0
HCS	9	12,9
CODH	5	7,1

^*∗*^Column percentage.

**Table 2:** Rate of correctly answering the questions in the sections based on sex, age, mean age, year, occupational group, and practice setting.

	Definition, risk factors, physiopathology, and epidemiology of COPD		Diagnosis and management of COPD		Basic concepts in pulmonary function test (PFT) for COPD		Patient training and pharmacological treatment in stable COPD		Nonpharmacological treatment in COPD		Approach to diagnosis and management in exacerbations of COPD	
	Section 1		Section 2		Section 3		Section 4		Section 5		Section 6	
	*n*	%	*n*	%	*n*	%	*n*	%	*n*	%	*n*	%
Sex												
Male	15	75,0^*∗*^	3	75,0	7	77,8	1	33,3	5	83,3	1	100,0
Female	5	25,0	1	25,0	2	22,2	2	66,7	1	16,7	0	0,0
Mean age												
Above mean age (>45,6)	16	72,7	4	66,7	3	33,3	1	25,0	5	71,4	0	0,0
Below mean age (<45,6)	6	27,3	2	33,3	6	66,7	3	75,0	2	28,6	1	100,0
Mean number of working years												
Above mean number of working years (>21,4)	17	77,3^*∗*^	4	66,7	2	25,0	1	33,3	4	57,1	0	0,0
Below mean number of working years (<21,4)	5	22,7	2	33,3	6	75,0	2	66,7	3	42,9	1	100,0
Occupational group												
Physician	5	20,8	1	16,7	1	11,1	1	25,0	0	0,0	0	0,0
Nonphysician healthcare provider	19	79,2	5	83,3	8	88,9	3	75,0	7	100,0	1	100,0
Practice setting												
FHC	19	82,6	4	66,7	7	77,8	3	75,0	7	100,0	1	50,0
HCS	4	17,4	0	0,0	0	0,0	1	25,0	0	0,0	0	0,0
CODH	0	0,0	2	33,3	2	22,2	0	0,0	0	0,0	1	50,0

^*∗*^ *p* < 0.05.

## Appendices

Assessment for COPD and pulmonary rehabilitation before education.

### A. Sociodemographical Characteristics

1. Your degree:
  - Practitioner
  - Specialist physician
  - Other……………………….
2. Gender:
  - Female
  - Male
3. Age: …………………
4. Your unit:
  - Family health center
  - Home-care services
  - Other……………………….
5. How long have you been working in the profession: for…………………………… years

### B. Questions about COPD and Pulmonary Rehabilitation

1. Which of the following is not a risk factor for COPD?
  - Exposure to tobacco smoke
  - Alpha-1 antitrypsin deficiency
  - Biomass exposure
  - Vitamin B12 deficiency
  - Low birth weight
2. What ranks COPD as a leading cause of death in Turkey?
  - 1
  - 2
  - 3
  - 4
  - 5
3. Which of the following is the criteria for obstruction in respiratory function test for COPD?
  - FEV1 < 70%
  - FVC < 70%
  - FEV1/FVC < 70%
  - PEF < 70%
  - FEV1 < 70% and FVC < 70%
4. Which of the following is correct about differential diagnosis of asthma and COPD?
  - Asthma beginning on more than 40 years
  - COPD being generally over 10-year history of cigarette packs
  - COPD being a progressive lung function but may be returning to normal
  - Developing asthma only by allergen exposure
  - Being often observed in the sputum of asthma
5. In a patient presenting with dyspnea and productive cough lasting for 5 years, which of the following is the method that should be performed first when airway obstruction has been found on pulmonary function test (PFT)?
  - Bronchodilator reversibility test
  - The bronchial provocation test
  - Chest X-ray
  - Complete blood analysis
  - Physical examination
6. Which of the following item/items may be primarily used to evaluate the response to the treatment of COPD patients?
  - FEV1/FVC
  - FEV1
  - IC
  - FVC
  - The flow-volume curve
7. Which of the following is the most significant intervention in COPD?
  - Quitting smoking and avoiding risk factors
  - Giving basic information on the disease
  - Teaching the principles of drug use and effective methods of inhalation
  - Prevention and early recognition of exacerbation
  - Oxygen treatment
8. Which of the following does not need to be inquired in all patients in COPD management?
  - Smoking cessation
  - Avoiding passive smoke exposure
  - Avoiding occupational dust exposure
  - Applying the influenza vaccine
  - Applying pneumococcal vaccine
9. Which of the following options is correct about the postbronchodilator predicted FEV1 evaluation in the management of COPD?
  - Pulmonary rehabilitation is started in the cases with FEV1 <80% of predicted value
  - Influenza and pneumococcal vaccines are applied in the subjects with FEV1 <50–80% of predicted value
  - Oxygen therapy is given in the subjects with FEV1 <50% of predicted value
  - Oxygen therapy is given in subjects with FEV1 <30% of predicted value
  - Surgical treatment is performed in the subjects with FEV1 <50% of predicted value
10. Which of the following* is not one* of the aims of the pulmonary rehabilitation in COPD?
  - Decreasing symptoms
  - Improving functional capacity
  - Improving quality of life
  - Reducing health-related expenses by reversing or stabilizing the systemic effects of the disease
  - Reducing the annual decline in FEV1
11. Which of the following* is not criteria for home-care* in COPD?
  - New diagnosis, comorbid disease
  - Any patient who cannot come to outpatient clinic but needing monitoring and/or education
  - Predicted FEV1 being less than 30%
  - Having more than one visit to the emergency service department or hospital admission over the last year
  - Use of long-term oxygen therapy
12. Which of the following* is not one of the headings* of evaluating effectivity in the pulmonary rehabilitation?
  - Exercise capacity
  - Quality of life
  - Degree of the symptoms
  - Body composition and psychosocial status
  - Chest X-ray findings
13. A patient who had had treatment for three times for exacerbation of COPD over the last year was presented to hospital with FVC of 60%, FEV1/FVC ratio of 55%, FEV1 45% of predicted and following values of arterial blood gases: pH: 7.35, PaO2: 55 mmHg, PaCO2: 50 mmHg, and SaO2: 88%. Which would be your approach to treatment of this patient?
  - Refer the patient to a center of higher level
  - Bronchodilator therapy with 3-4 L/min oxygen inhalation therapy is given
  - Oxygen treatment + 1 mg/kg of oral steroid is added to bronchodilator treatment
  - Inhaled bronchodilators are started with intervals of 4 hours
  - In addition to oral steroids bronchodilator + oxygen therapy, noninvasive ventilation is initiated
14. Which of the following is wrong regarding the use of antibiotics in exacerbation?
  - True rate of finding atypical bacteria is 5 to 10% although they are serologically detected during exacerbation
  - Amoxicillin should be given in the case of sputum culture revealing penicillin-sensitive* S. pneumonia* or non-beta lactamase producing bacteria
  - The macrolides are not effective against* H. influenza*
  - Cefuroxime axetil and cefprozil are the second-generation antibiotics most effective against* H. influenza*
  - Fluoroquinolones are the first option to be given for intermediate exacerbations to the patients using beta-lactam antibiotics over the last three months or having penicillin allergy
